# Comorbidity of attention deficit hyperactivity disorder in a patient with epilepsy: Staring down the challenge of inattention versus nonconvulsive seizures

**DOI:** 10.1016/j.ebr.2024.100651

**Published:** 2024-02-01

**Authors:** Derryl J. Miller, Hannah Komanapalli, David W. Dunn

**Affiliations:** a Clinical Neurology, Indiana University School of Medicine and Riley Hospital for Children, 705 Riley Hospital Dr, Indianapolis, IN 46202, USA; b Undergraduate Medical Education, Indiana University School of Medicine, 635 Barnhill Dr, Indianapolis, IN 46202, USA; c Psychiatry and Neurology, Indiana University School of Medicine and Riley Hospital for Children, 705 Riley Hospital Dr, Indianapolis, IN 46202, USA

**Keywords:** Attention deficit hyperactivity disorder, Epilepsy, Nonepileptic, Inattentiveness, Polypharmacy

## Abstract

Epilepsy is a heterogeneous disorder of recurrent seizures which often is comorbid with anxiety, depression, attention deficit hyperactivity disorder (ADHD), intellectual disability (ID), and other psychiatric manifestations. Treating both epilepsy and behavioral symptoms from psychiatric disorders can result in polypharmacy with interactions of medications leading to both worsened efficacy of antiseizure medications due to psychotropic effects and worsening of psychiatric symptoms due to antiseizure medication side effects. We aim to suggest pragmatic strategies for the neurologist in the diagnosis and management of comorbid ADHD in patients with epilepsy based on the International League Against Epilepsy (ILAE) Pediatric Commission guidelines and additional literature review. The screening tool of choice for the symptoms of ADHD is validated in the country of practice and written in the language of the family, though various screening tools and advantages and disadvantages of each will be discussed. Once ADHD is diagnosed, recent safety data suggest that Methylphenidate, Amphetamine, and Atomoxetine are generally safe for patients with epilepsy. We present a case of a child with epilepsy and ADHD and discuss the clinical signs, symptoms, and strategies for treatment as well as when to refer to child psychiatry.

## Highlights

• The ideal screener for ADHD is validated & translated into the family’s language.
• The Strengths & Difficulties Questionnaire screens for ADHD symptoms in 40 languages.
• ADHD signs include inattentive symptoms and hyperactivity symptoms over 6 months.
• Methylphenidate, amphetamine, & atomoxetine are used safely in epilepsy patients.
• Psychiatry should evaluate patients without response to first line agents for ADHD.

## Case study

A 16 year old boy with combined ADHD, migraine with aura, tic disorder, and generalized epilepsy was maintained on combination of stimulants and antiseizure medications. He had history of generalized tonic clonic seizures and absence seizures. He had previously been trialed on oxcarbazepine which worsened his staring episodes. He also was briefly placed on low dose of topiramate for his migraines, though his seizures incompletely resolved and his headaches overall improved with lifestyle modifications so topiramate was stopped. His birth was unremarkable, and he had no history of significant head trauma, intracranial surgery, or meningoencephalitis. He did have developmental delay with late walking at 18 months and delayed speech of 3.5 years old. His brother had focal seizures following head trauma, and his grandmother has seizures and lupus. His seizures were successfully treated with Valproate, and overnight video electroencephalogram (EEG) monitoring suggested seizure freedom even with activation maneuvers with photic stimulation and hyperventilation. His EEG showed generalized interictal epileptiform discharges placing him at risk for generalized onset seizures, and magnetic resonance imaging (MRI) of the brain without contrast was unremarkable. He was noted to have a poor attention span with impulsivity and hyperactivity despite being seizure free, and, as his behavioral symptoms were incompletely improved with Valproate, he was screened for comorbidities. Given the clinician’s comfort with the available screeners for ADHD, the National Institute for Children’s Health Quality (NICHQ) Vanderbilt Assessment Scale was filled out by his teacher and father with diagnosis of combined type ADHD [1], [2]. The NICHQ Vanderbilt Assessment Scale is structured with the first 9 questions being inattentive symptoms, the second 9 questions being hyperactive symptoms, and screening questions for oppositional defiant disorder, conduct disorder, and anxiety and depression that follow [2]. Furthermore, diagnosis of ADHD requires a school performance score which is elevated, suggesting that the patient is having academic difficulties due to symptoms of ADHD [2]. The teacher Vanderbilt Assessment had 8 positive inattentive symptoms and 7 positive hyperactive symptoms on his initial screening with total symptom score of 40 and multiple performance questions with score over 4, while his father’s Vanderbilt Assessment showed 6 positive inattentive symptoms and 5 hyperactive symptoms with total symptom score of 27 with no positive performance score but with overall school performance at a 3. He was trialed on Lisdexamfetamine with significant improvement in both attention and hyperactivity at school.

He presented to neurology clinic with continued staring episodes concerning for possible seizures versus inattentiveness on Lisdexamfetamine 30 mg PO daily, and he was advised to stop Lisdexamfetamine due to the risk of lowering his seizure threshold. Following discontinuation of Lisdexamfetamine, he had significant troubles with attention and with hyperactive behaviors at school. His school determined that his behavior was significantly worse, and the mother, thinking this could be due to his Valproate, stopped this as well. His behavior did not improve with stopping Valproate. He had no anxiety, no depression, no suicidal ideation, and no homicidal ideation. He did not have self-harm behaviors. Upon further discussion, his academics were more difficult in the 10th grade following discontinuation of Lisdexamfetamine, so Lisdexamfetamine and Valproate were both restarted with improved behavior and academic performance in school. He remained seizure free per report in clinic which was confirmed with a follow up 2 day VEEG after restarting both medications. He has a 504 plan through the school for accommodations for his migraines and for epilepsy.

## Introduction

### Diagnosis and comorbidity of epilepsy with attention deficit hyperactivity Disorder

Attention deficit hyperactivity disorder (ADHD) is a well classified neurodevelopmental disorder in the Diagnostic and Statistical Manual of Mental Disorders, Fifth Edition (DSM-5) with 4 subtypes including Inattentive, Hyperactive/Impulsive, Combined, and Unspecified types [3]. In children and adolescents, six symptoms of inattention and/or hyperactivity must be present for over 6 months and cause disruption or be developmentally inappropriate for the diagnosis [3]. The prevalence of ADHD is approximately 5 % in children and 2.5 % of adults [3].

Functional MRI suggests an underlying predisposition to ADHD in patients with childhood absence epilepsy as demonstrated by reduced attention network activity in the anterior insula, medial frontal cortex, and frontal operculum even when seizures were well controlled, as demonstrated with EEG [4]. Our patient had generalized epilepsy with multiple generalized tonic clonic seizures and absence seizures and was diagnosed with combined type ADHD [5], [6], [7], [8].

Further confounding the diagnosis of comorbid ADHD and epilepsy, the semiology of generalized onset seizures and focal impaired awareness seizures involve transient loss of awareness which can mimic inattentiveness. The cognitive effects of antiseizure medications on patients with epilepsy may also worsen inattentiveness, making the clinician likely to consider iatrogenic cause rather than diagnosis of comorbid ADHD in children with epilepsy [9]. There is also a 2.5 times association of seizures in children with ADHD when compared to age and sex matched controls without history of ADHD in one cohort study [10]. An additional cohort study first identifying patients with ADHD who develop epilepsy showed a hazard ratio of 2.54 compared to healthy controls, and patients with epilepsy who develop ADHD had a hazard ratio of 3.24 compared to healthy controls [11]. The bidirectional association of ADHD and epilepsy suggests that these disorders may share common causes due to high comorbidity [10], [11]. Due to a bidirectional association of epilepsy and ADHD, annual screening for ADHD in children with epilepsy is advised and consideration of epilepsy in children with diagnosis of ADHD who appear intractable to stimulants and nonstimulants is appropriate [9], [10], [11], [12], [13], [14].

Due the high comorbidity of ADHD with epilepsy, the ILAE Pediatric Commission produced evidence-based guidance for the screening, diagnosis, and management of children with comorbid epilepsy and ADHD [9]. The screening tool of choice by the ILAE Pediatric Commission task force is a questionnaire written in the language of the patient and family, and the Strengths and Difficulties Questionnaire has been utilized often as it has been translated into over 40 languages and is free [9], [15]. The population of patients with ADHD with comorbid epilepsy are demographically different than those with ADHD without epilepsy as there is no predilection to male or female sex, and the ADHD subtype is predominantly inattentive presentation more commonly than combined presentation as seen in psychiatric clinics [9]. In the general population of patients without epilepsy, ADHD is more highly prevalent in boys [9]. About 30–40 % of patients with epilepsy have comorbid inattentive ADHD [3], [9]. Further complicating the diagnosis of ADHD in patients with epilepsy is the high incidence of learning disabilities in patients with epilepsy such that neuropsychological testing should be considered to rule out comorbid learning disability.

Our patient was diagnosed with combined inattentive and hyperactive ADHD via NICHQ Vanderbilt Assessment Scales by both a parent and a teacher. The NICHQ Vanderbilt Assessment Scale is validated for children ages 6–12 years old and provides discriminate diagnosis of inattentive ADHD, Hyperactive/Impulsive ADHD, and combined type ADHD with additional screening questions for Oppositional Defiant Disorder (ODD), Conduct Disorder, Anxiety, and Depression. The NICHQ Vanderbilt Assessment Scale is commonly used in the United States as it is validated and in English. Additional screening tools for ADHD include the Conners 3 ADHD Index [16], the ADHD Diagnostic Rating Parent Scale [17], and broad-based questionnaires include the Strengths and Difficulties Questionnaire [15] which is translated into many languages and is free, and the Pediatric Symptom Checklist [18], which is also free. The advantages of ADHD specific screeners such as the ADHD Rating Scale, Swanson, Nolan and Pelham (SNAP) Questionnaire, and Wender Utah Rating Scale is that these are brief and easy to administer and score with most questions involving symptoms of ADHD [20], [21], [22]. More broad-based questionnaires are longer and have more complex scoring with only some questions pertaining to symptoms of ADHD such as the Strengths and Difficulties Questionnaire or NICHQ Vanderbilt Assessment Scale [2], [15]. Other advantages to broad-based screeners would be the potential detection of other comorbidities such as anxiety and depression which will be discussed in other articles in this special issue. There is currently no specific screener validated in a population of youth with epilepsy for ADHD [19]. A list of available screening tools for ADHD is noted in Table 1 [2], [15], [16], [20], [21], [22].

**Table 1:** Screeners available for assessing symptoms of ADHD with both broad-based and ADHD specific questionnaires.

Screener Name	Validated Ages	# of Items	Conditions Tested	Cost
NICHQ Vanderbilt Assessment Scale Diagnostic Rating Scale	6–12 years	55	ADHD, conduct disorder, oppositional-defiant disorder, anxiety, depression	Free
ADHD Rating Scale IV – Preschool Edition	3–5 years	18	ADHD	Free
Swanson, Nolan, and Pelham (SNAP) Questionnaire – IV	6–18 years	18	ADHD	Free
Wender Utah Rating Scale	>18 years	25	ADHD	Free
Strengths and Difficulties Questionnaire	2–18 years	25	ADHD, Conduct Problems, Mood disorders, Peer Relationship Problems, Prosocial Behavior	Free
Conners Rating Scale 3rd Edition	6–18 years	10	ADHD, Learning Problems, Executive Functioning, Defiance/Aggression, Peer Relations	Manual $147 & 25 print forms $105
Conners 3 ADHD Index	6–18 years	10	ADHD	Free

The differential diagnosis for inattentiveness poses a unique challenge to both neurologists and psychiatrists due to the broad possible diagnoses for patients with paroxysmal staring. Among this differential is nonmotor seizures, sleep disorders resulting in daytime sleepiness, poor social reciprocity as is seen with autism spectrum disorder or other cognitive disorders, hearing loss, underlying genetic disorders, intellectual disability, static encephalopathies due to perinatal insults, other psychiatric comorbidities such as anxiety and depression, iatrogenic polypharmacy, and other causes of inattentiveness such as ADHD [23], [24]. Furthermore, if comorbidities potentially mimicking ADHD are present, it is likely that patients could screen positive on an ADHD screening test so clinicians must have a high index of suspicion for inattentiveness being related to incompletely treated comorbidities and consider a broad workup if the history and physical exam are suggestive or if the patient does not respond to conventional therapies for ADHD [25].

### Pharmacologic treatment considerations

#### Psychotropics to treat ADHD

The ILAE Pediatric Commission Taskforce has noted that many neurologists are reluctant to prescribe agents to treat ADHD due to concerns that such medications may lower the seizure threshold [9]. An increasing body of literature with large cohorts is suggestive that ADHD medications are not likely to exacerbate seizures [9], [26], [27], [28]. Two large retrospective cohorts showed increased risk of seizures in patients with ADHD compared to those without ADHD, though no increased risk of seizures longitudinally within-individual comparisons following treatment of ADHD in those individuals with ADHD [27], [28]. In fact, both retrospective cohorts found that the use of ADHD medications was associated with decreased occurrence of seizures during periods of ADHD medication use even if patients had a prior history of seizures [27], [28]. An evidence-based review of the literature by the ILAE Pediatric Commission Taskforce resulted in recommendations on the safety of psychotropics in patients with epilepsy, including Methylphenidate, Amphetamine, and Atomoxetine in 2018 [9]. Methylphenidate is felt most safe due to the available evidence including blinded trials in children with epilepsy [9], [29]. There have been no head-to-head trials of Methylphenidate and Amphetamine in children with epilepsy and both stimulants are likely effective and safe for children with comorbid ADHD and epilepsy.

Methylphenidate is a stimulant medication indicated in randomized controlled trials as being unlikely to exacerbate seizures (0–18%) and provides significant symptom relief over 2/3 of the time if titrated appropriately [9]. Methylphenidate immediate release is started at 2.5–5 mg PO twice daily and increased by 5–10 mg per dose titrated weekly to a maximum dose of 2 mg/kg/day or 60 mg/day [30]. Treatment failure is generally known within 1 week due to short half-life. Methylphenidate also comes as an extended-release formulation dosed daily or twice daily starting at 10 mg PO daily and increasing 10 mg/day every week to maximum dose of 2 mg/kg/day [30]. The Osmotic Release Oral System (OROS) Methylphenidate is a long-acting formulation with advanced controlled release via a semipermeable membrane tablet affording once daily dosing of up to 2 mg/kg/day or 72 mg per day [30], [31]. OROS Methylphenidate can also be considered in cases where there is a concern for abuse or diversion. One advantage of Methylphenidate for the child neurologist to consider includes the availability of multiple formulations including tablets, chewable pills, liquids, and skin patches and preparations with varying duration of action including short-acting (4 h), intermediate (8 h), and long-acting (10–12 h) Methylphenidate. Common adverse reactions of Methylphenidate include decreased appetite, dry mouth, nausea, headaches, insomnia, and irritability [30]. Monitoring on Methylphenidate includes assessing for personal or family history of arrhythmia or cardiac disease and assessing with EKG and echocardiogram in such cases as well as monitoring appetite, growth, complete blood count (CBC), blood pressure, and heart rate [30].

Amphetamine is probably equally effective and safe for treatment of ADHD in people with epilepsy though there is less data from controlled trials. Amphetamine was studied in an open label retrospective uncontrolled design in patients under 18 years old with aim to determine if Methylphenidate or Amphetamine were more likely to help symptoms of ADHD without worsening seizures [32]. In this study, Methylphenidate outperformed Amphetamine in response of 62% versus 37%, and Methylphenidate only worsened seizures in 5% of patients while Amphetamine worsened seizures in 11% of patients [32]. As previously noted, large cohorts since this recommendation utilizing within-individual analysis showed no increase in seizures with Methylphenidate [27], [28]. Amphetamine is furthermore considered first line in adults with epilepsy and ADHD for safety and efficacy [29]. Amphetamine is started at 5 mg PO daily or twice daily and increased every 7 days by 5 mg/day to 40 mg/day, though an upper limit of dosing is not specified [33]. Amphetamine formulations also are available as tablets, chewable pills, and liquids and both short (4–5 h) and long (10–12 h) preparations. Treatment failure is known within 1 week due to short half-life. The most common adverse reactions to amphetamine include abdominal pain, decreased appetite, insomnia, and emotional lability [33]. Monitoring for Amphetamine includes assessing for personal or family history of arrhythmia or cardiac disease and sending EKG and echocardiogram in such cases as well as monitoring appetite, growth, complete blood count (CBC), liver enzymes (AST/ALT), blood pressure, and heart rate [33].

Nonstimulant medication such as atomoxetine are also unlikely to exacerbate seizures (7 %) though the evidence for this is more limited [9]. Atomoxetine is started at 0.5 mg/kg/day orally and increased after one week to maximum dose of 1.2 mg/kg/day or 100 mg/day [34]. For patients who are over 70 kg, atomoxetine is started at 40 mg/day and increased up to 100 mg/day [34]. Treatment failure is also known within three weeks. Common adverse effects include increased blood pressure and heart rate, hyperhidrosis, abdominal pain, nausea, dry mouth, drowsiness, headache, insomnia, and erectile dysfunction [34]. Monitoring for atomoxetine includes assessing for personal or family history of arrhythmia or cardiac death and assessing with EKG and echocardiogram in such cases along with monitoring appetite, growth, liver enzymes (AST/ALT), blood pressure and heart rate [34]. Other nonstimulants approved in the United States for treatment of ADHD include guanfacine ER, clonidine ER, and viloxazine. These agents have not had trials in people with epilepsy and ADHD. ADHD preparations recommended by the ILAE Pediatric Commission are summarized in Table 2.

**Table 2:** Pharmacotherapy available and recommended for treatment of behavioral symptoms of ADHD recommended by the ILAE Pediatric Commission task force as well as their doses (9).

ADHD Medication	Starting Dose	Half Life	Steady State	Maximum Dose
Methylphenidate	2.5–5 mg PO twice daily	2.5 Hr (IR), 3.4–6.8 Hr (ER)	12.5 Hr (IR), 34 Hr (ER)	2 mg/kg/d or 60 mg/d
Methylphenidate OROS	18 mg PO daily	12 Hr (controlled release)	60 Hr (controlled release)	2 mg/kg/d or 72 mg/d
Amphetamine	5 mg PO daily or twice daily	9–14 Hr	70 Hr	40 mg/d
Atomoxetine	0.5 mg/kg/day PO	5.2 Hr	26 Hr	1.2 mg/kg/d or 100 mg/d

Our patient was successfully treated for his combined presentation ADHD with Lisdexamfetamine which is a prodrug of d-amphetamine [35]. There is insufficient evidence to recommend Lisdexamfetamine as treatment before Methylphenidate or Adderall, though previous cohorts have shown that Methylphenidate ER was noninferior to Lisdexamfetamine [36]. Additionally, the use of Lisdexamfetamine is considered in the National Institute for Health and Care Excellence (NICE) guideline though was not recommended by the recent ILAE Pediatric Commission taskforce [9], [37].

#### Antiseizure medications

If comorbid diagnoses of ADHD and epilepsy are encountered, several pharmacologic agents can be utilized for the patient’s seizures and inattentive or hyperactive symptoms, though polypharmacy and side effect profiles must be considered [25], [38], [39], [40], [41]. Children with ADHD furthermore can have associated anxiety, depression, and impulsive aggression, necessitating care in the selection of antiseizure medications to reduce exacerbation of comorbid disorders of behavior which will be discussed further in other articles of this special issue [42], [43], [44]. A meta-analysis of randomized controlled trials and cohort studies indicated that several antiseizure medications have been noted to worsen ADHD symptoms such as Phenobarbital, Phenytoin, Ethosuximide, Zonisamide, Perampanel, Topiramate, and Valproate [26]. A few antiseizure medications have been found to have positive effects on ADHD symptoms such as Carbamazepine and Lamotrigine [26]. At times, antiseizure medications can worsen comorbid psychiatric disorders with ADHD resulting in aggressive behaviors, suicidal ideation, and homicidal ideation, such as Levetiracetam, Perampanel, and Topiramate [45], [46]. In children and adolescents, Levetiracetam was statistically more commonly associated with psychiatric side effects and behavioral side effects including depression, anxiety, irritability, and tantrums in a cross-sectional study compared with Lamotrigine, Valproate, Topiramate, and Carbamazepine [47]. These behavioral side effects of Levetiracetam were also dose dependent, and symptoms often resolved with dose reduction [47]. Lacosamide and Carbamazepine have been shown to have lower incidence of aggressive behaviors compared to Levetiracetam in adults in a prospective cross-sectional study of self-reported symptoms utilizing the Bus Perry Aggression Questionnaire [48]. When significant behavioral side effects are encountered with Levetiracetam, several cohorts of both children and adults have demonstrated efficacy of Pyridoxine (vitamin B6) in reducing psychobehavioral symptoms [49], [50], [51]. Many antiseizure medications do not have sufficient data for or against exacerbation of symptoms of ADHD which may prompt additional study such as the following: Cenobamate, Retigabine, Stiripentol, and Tiagabine [25], [52], [53], [54], [55]. Our findings based on this literature review are summarized in Table 3.

**Table 3:** Results of literature search examining psychobehavioral and cognitive effects on ADHD symptoms of antiseizure medication.

Positive Effect	Neutral	Negative Effect	Unknown/Study Needed
Carbamazepine	Brivaracetam	Phenobarbital	Cenobamate
Clobazam	Cannabidiol	Phenytoin	Retigabine
Lamotrigine	Clonazepam	Ethosuximide	Stiripentol
	Eslicarbazepine	Topiramate	Tiagabine
	Felbamate	Zonisamide	
	Gabapentin	Valproic Acid	
	Oxcarbazepine	Perampanel	
	Primidone	Levetiracetam	
	Rufinamide	Vigabatrin	

In patients first diagnosed with epilepsy and without comorbid inattentiveness or hyperactivity, screening for ADHD should also be performed at intervals, especially after antiseizure medications are started (9,47). Screenings should be performed at least annually and utilizing a validated screening tool in the family and child’s primary language as recommended by the ILAE Pediatric Commission [9]. Children of mothers with epilepsy who require antiseizure therapy during gestation are at increased risk for neurocognitive delays and should be screened at intervals, especially if the mother was maintained on Valproate, though there is conflicting evidence to support that Valproate exposure in utero increases risk of ADHD in children [56], [57].

Following diagnosis of comorbid ADHD and treatment with appropriate agents, response to treatment may be assessed several ways. In the case of the NICHQ Vanderbilt Scale, there are initial questionnaires and follow up questionnaires for parents and teachers which can be followed to assess response [2]. Additionally, neurologists may perform a within-individual comparison with the diagnostic screener of choice. Finally, assessment of school academic performance and family’s report of symptoms also can be helpful in assessing response to therapy.

While antiseizure medications are known to exacerbate behavioral side effects as previously discussed, multiple classes of other medications are known to exacerbate behavioral side effects [58]. Neurologists and psychiatrists caring for patients with medically complex history must review and simplify medication regimens as appropriate if iatrogenic cause of behavioral side effects is suspected. Due to worsening of seizures or behaviors with polypharmacy, nonpharmacologic interventions for epilepsy with ADHD could be considered with the goal of reducing side effects and number of antiseizure medications such as the ketogenic diet or epilepsy surgeries in the case of medically intractable epilepsy.

#### Medication interactions

The treatment of comorbid epilepsy and ADHD results in polypharmacy, and interactions between psychotropic medications and antiseizure medications must be considered. Cannabidiol, used in several epilepsy syndromes including Lennox-Gastaut syndrome, Dravet syndrome, and Tuberous sclerosis, has been associated with an increase in Methylphenidate concentrations, and Phenobarbital concentrations are increased in patients taking Methylphenidate [30]. When Amphetamine is given with Cannabidiol, this may worsen tachycardia [33]. Carbonic anhydrase inhibitors such as Topiramate and Acetazolamide may decrease excretion of Amphetamine [33]. Atomoxetine is noted to have minimal interaction with antiseizure medications [34].

### When to refer to psychiatry

Due to paucity of access to subspecialty healthcare providers, patients with anxiety, depression, and ADHD are often diagnosed and treated by primary care providers. In the case of patients with epilepsy who are closely managed by a neurologist, many comorbid cases of ADHD will be encountered and can be treated by the neurologist. The ILAE Pediatric Commission has suggested first line therapies for treatment of comorbid ADHD to be Methylphenidate, Amphetamine, or Atomoxetine with the most data favoring Methylphenidate as first line therapy. In the cases of difficulty confirming the diagnosis of ADHD, ruling out other comorbid DSM5 diagnoses such as bipolar disorder or psychosis, or failure of the patient to respond to first line agents, referral to psychiatry for evaluation and treatment is advised.

## Summary

ADHD and epilepsy are encountered with high comorbidity due to bidirectional association. Because inattentiveness includes a broad differential, early testing to narrow the differential and to diagnose ADHD is advised with screening tools which can be either selective to diagnose ADHD or broad-based screening to detect co-morbid psychiatric disorders which will be further discussed elsewhere in this special issue. The most appropriate screener for the neurologist for diagnosis of comorbid ADHD remains a screener validated in the country of practice and ideally in the patient and family’s language. If comorbid ADHD and epilepsy are encountered, clinicians will need to treat inattentiveness and hyperactivity while also prescribing antiseizure medications which lend themselves to minimal behavioral or cognitive side effects. The body of literature now suggests that treatment of ADHD in children with epilepsy can safely be accomplished in most cases with Methylphenidate, Amphetamine, or Atomoxetine with the best evidence for efficacy and safety for Methylphenidate.

## CRediT authorship contribution statement

**Derryl J. Miller:** Writing – original draft, Writing – review & editing, Supervision, Conceptualization. **Hannah Komanapalli:** Writing – review & editing. **David W. Dunn:** Writing – review & editing, Supervision, Conceptualization.

## Declaration of competing interest

The authors declare that they have no known competing financial interests or personal relationships that could have appeared to influence the work reported in this paper.
